# Seroreactivity for spotted fever rickettsiae and co-infections with other tick-borne agents among habitants in central and southern Sweden

**DOI:** 10.1007/s10096-012-1742-3

**Published:** 2012-09-09

**Authors:** A. Lindblom, K. Wallménius, M. Nordberg, P. Forsberg, I. Eliasson, C. Påhlson, K. Nilsson

**Affiliations:** 1 Unit of Infectious Diseases, Department of Medical Sciences, Uppsala University, Uppsala, Sweden; 2 Unit of Clinical Bacteriology, Department of Medical Sciences, Uppsala University, 751 85 Uppsala, Sweden; 3 Unit of Infectious Diseases, Department of Clinical and Experimental Medicine, Faculty of Health Sciences, Linköping University, Linköping, Sweden; 4 Department of Infectious Diseases, County Council of Östergötland, Linköping, Sweden; 5 Department of Laboratory Medicine, Norra Älvsborg County Hospital (NÄL), Trollhättan, Sweden; 6 Center of Clinical Research, Falun, Dalarna Sweden

## Abstract

Patients seeking medical care with erythema migrans or flu-like symptoms after suspected or observed tick bite in the southeast of Sweden and previously investigated for *Borrelia* spp. and/or *Anaplasma* sp. were retrospectively examined for serological evidence of rickettsial infection (Study 1). Twenty of 206 patients had IgG and/or IgM antibodies to *Rickettsia* spp. equal to or higher than the cut-off titre of 1:64. Seven of these 20 patients showed seroconversion indicative of recent or current infection and 13 patients had titres compatible with past infection, of which five patients were judged as probable infection. Of 19 patients with medical records, 11 were positive for *Borrelia* spp. as well, and for *Anaplasma* sp., one was judged as positive. Five of the 19 patients had antibodies against all three pathogens. Erythema migrans or rash was observed at all combinations of seroreactivity, with symptoms including fever, muscle pain, headache and respiratory problems. The results were compared by screening an additional 159 patients (Study 2) primarily sampled for the analysis of *Borrelia* spp. or *Mycoplasma pneumoniae*. Sixteen of these patients were seroreactive for *Rickettsia* spp., of which five were judged as recent or current infection. Symptoms of arthritis, fever, cough and rash were predominant. In 80 blood donors without clinical symptoms, approximately 1 % were seroreactive for *Rickettsia* spp., interpreted as past infection. The study shows that both single and co-infections do occur, which illustrate the complexity in the clinical picture and a need for further studies to fully understand how these patients should best be treated.

## Introduction

Tick-transmitted diseases are an emerging health problem in temperate regions of the northern hemisphere. The hard tick *Ixodes ricinus* is the main European vector for agents such as *Borrelia burgdorferi*, *Anaplasma phagocytophilum*, tick-borne encephalitis (TBE) virus and most of the species of the spotted fever group of rickettsiae (SFG), of which at least nine are recognised pathogens in humans in this part of the world [1, 2].

 In Sweden, predominantly *Rickettsia helvetica*, at a variable prevalence of 1.7–17.3 % and in one case also *Rickettsia sibirica*, have been detected in *I. ricinus* ticks gathered from several parts of the country [3, 4]. Infections with *R. helvetica* are usually recognised as a mild febrile illness, but more severe symptoms such as perimyocarditis and meningitis have been reported [5–10]. Another rickettsia, *R. felis*, whose main reservoir and vector is cat fleas (*Ctenocephalides felis*), has, so far, not been reported in any vector in Sweden, although it has been shown to cause meningitis in two cases [11].

 Ticks may be simultaneously co-infected with multiple agents [3, 12]. However, co-infections in humans of multiple tick-borne agents have rarely been studied [13]. The aim of the present project was to examine the serological evidence of rickettsial infections in a population naturally exposed to ticks, the characteristic symptoms of the rickettsial disease and to what extent rickettsial co-infections with other tick-borne agents occur in Sweden.

 The present report is a retrospective independent part of a previous investigation (Study 1) examining serological responses to several vector-borne agents in the same serum material, in which the presence of antibodies to *Borrelia* spp., *Anaplasma* sp. and TBE virus has been reported separately [14]. It includes also an additional serological examination (Study 2) of patients primarily sampled for possible borreliosis as well as analysed for the presence of antibodies to *Mycoplasma* spp.

## Patients and methods

### Patients and sera of Study 1

Sera were obtained from 206 patients seeking medical care from May to December 2001 with flu-like symptoms or erythema migrans (EM) after suspected or observed tick bite in southeastern Sweden. The patients were between the ages of 16 and 87 years, 110 (53 %) patients were females and 96 (47 %) patients were males. Each patient enrolled in the study was sampled for three sera (S1–S3); enrolment day 0 (S1), sample 2 (S2) collected 6–8 weeks after enrolment and a third sample (S3) 6 months after enrolment. All sera were stored in the freezer for later analysis. Two of these sera, S1 and S2, from all 206 patients were analysed for rickettsial antibodies. Initially, sample number 2 (S2) for all 206 serum samples were screened for IgG antibodies to *Rickettsia* spp. Samples with a titre equal to or higher than the cut-off titre of 1:64 were re-tested for IgM (S2). The corresponding patient sample number 1 (S1) was, thereafter, examined in the same manner for IgG and IgM antibodies against *Rickettsia* spp. S3 was not used in this study. Data on symptoms and laboratory data were obtained from medical records based on the initial examination and subsequent follow-up interview 6–8 weeks after enrolment. Patient no. 1(Table 2) died during the study, which is why data other than those reported are missing. Prior to our study, the sera had been analysed for antibodies against *Borrelia* spp., *Anaplasma* sp. and tick-borne encephalitis (TBE) virus. Among these 206 patients, 186 patients with Lyme borreliosis (LB) were found (174 with EM), 18 with confirmed and two with probable human granulocytic anaplasmosis (HGA) and two cases of TBE [14].

### Patients and sera of Study 2

A total of 112 patients who, regardless of indication, submitted samples for analysis of LB and 47 patients analysed for *Mycoplasma pneumoniae* at Uppsala University Hospital during the period March–April 2012 were examined also for the presence of rickettsial antibodies, in the same way as in Study 1. Twenty-eight of 112 patients had serological signs of actual or previous exposure to LB and 11 of 47 patients to *M. pneumoniae*.

 As a control group, sera from 80 healthy blood donors were tested for rickettsial antibodies in the same manner.

### Methods

#### Immunofluorescence assay (IFA)

Antigen prepared from an aliquot of *R. helvetica*-infected Vero cells was supplemented with 10 % yolk sac solution and applied to microscope slide wells, dried, fixed in acetone and incubated with serial dilutions of serum, as previously described [15]. As positive controls, a serum sample from a patient with proven end-point IgG and IgM titres of 1:80 and 1:160, respectively, to *R. helvetica* and a serum sample from a patient with proven infection with *Rickettsia conorii* with end-point IgG titres of 1:160, provided by the Swedish Institute for Infectious Disease Control (SMI), were used. Phosphate buffered saline was used as negative control and all positive samples were re-tested with human blood donor serum as negative control. IgG and IgM antibodies were detected by specific polyclonal rabbit anti-human fluorescein isothiocyanate-conjugated (FITC) γ (IgG) and Mu-chain (IgM) conjugated antibodies (refs.: F0202 and F0203; Dako, Glostrup, Denmark). The IgM antibodies were examined after a pre-treatment procedure with rheumatoid factor adsorbent (Immunkemi, Stockholm, Sweden) to remove complex bound IgG antibodies. For IgG, a titre ≥1:64 but <1:256 was considered a past infection and >1:256 a recent or current infection, while <1:64 was regarded as negative. For IgM, a titre <1:64 was considered to be negative and >1:64 a recent or current infection. A probable infection was defined as a 4-fold increase in IgG antibody titre between S1 and S2 and with 1:128 as the highest measured titre in S2.

#### Western blot (WB)

Sera at a dilution pf 1:400 from three of the IgG-positive patients in Study 1 (nos. 6, 14 and 15) was tested for WB. As the antigen, a 1,401 bp cloned DNA fragment of the *omp*B gene of *R. helvetica*, amplified in the polymerase chain reaction (PCR) assay, was ligated into a pET102/D-TOPO vector and expressed in the BL21 component *Escherichia coli* following the manufacturer’s instructions (TOPO TA Cloning Kit for Sequencing, Invitrogen) as a fusion protein of 61 kDa.

The 1,401 bp cloned DNA sequence of the *omp*B gene corresponding to a 467 amino acid peptide of the native protein (aa 761–1227) was amplified by primers chosen for *R. helvetica* with the sequences (Rh-ompB-F) 5′CACACAATCTGCCGATAATACCGG and (Rh-ompB-R) 5′TACACCAGGTGCACCTCCA. The thermal cycle conditions for PCR of the *omp*B gene of the rickettsiae have been described previously [3]. The conventional PCR assays were performed in a GeneAmp® PCR System 9700 (Applied Biosystems, Foster City, CA, USA). Amplified PCR products were separated by electrophoresis on a 1 % agarose gel stained with Gel Red™ (Biotium), illuminated by UV light and compared with the DNA molecular weight marker GeneRuler™ Express DNA ladder (Fermentas GmbH, St. Leon-Rot, Germany). The purified peptide was dissolved in Laemmli solution and transferred into nitrocellulose membrane (Bio-Rad), blocked with 5 % non-fat dried milk and overlaid with serum, incubated, washed and incubated with horseradish peroxidase (HRP)-conjugated goat anti-human IgG (Bio-Rad, Goat-anti-Rabbit, cat no. 172–1050), as previously described [16]. A serum from rabbit immunised with purified *R. helvetica* was used as the positive control, and the secondary antibody alone served as the negative control.

#### Methods used in previous analyses of Study 1

*B. burgdorferi* IgG and IgM were examined using a commercial enzyme-linked immunosorbent assay (ELISA) according to the manufacturer’s instructions for use and interpretation (Genzyme Virotech GmbH, Rüsselsheim, Germany). Positive or equivocal samples from ELISA were further tested by WB (Genzyme Virotech GmbH, Rüsselsheim, Germany), using an Autoblot 36 (Genelabs Diagnostics, Irvine, CA, USA). The patients were considered to be positive based on seroconversion, a significant rise in IgG titre and/or appearance of new significant bands in the WB banding pattern between S1 and S2. IgG antibodies (S1 and S2) to *A. phagocytophilum* were detected using a commercial kit (Focus Technologies, Cypress, CA, USA). A titre >1:80 was considered to be positive and laboratory evidence of infection was based on seroconversion or a >4-fold rise in titre between S1 and S2. A probable infection was defined as a permanently high IgG antibody titre of ≥1:640 or at least a 4-fold decrease in IgG antibody titre during the investigation period. Infection with TBE virus was based on positive IgM screen (S1) [Immunozym FSME (Frühsommer-Meningoenzephalitis) IgM or Progen Biotechnik GmbH, Germany] and confirmed by the rapid fluorescent focus inhibition test (RFFIT) [14, 17].

####  Methods used in previous analyses of Study 2

All sera were analysed for IgG and IgM antibodies against *Borrelia* spp. using the Euroimmun’s ELISA kit [Euroimmun AG (Aktiengesellschaft), Lübeck, Germany], according to the manufacturer’s instructions.

## Results

### Study 1

**Fig. 1:**
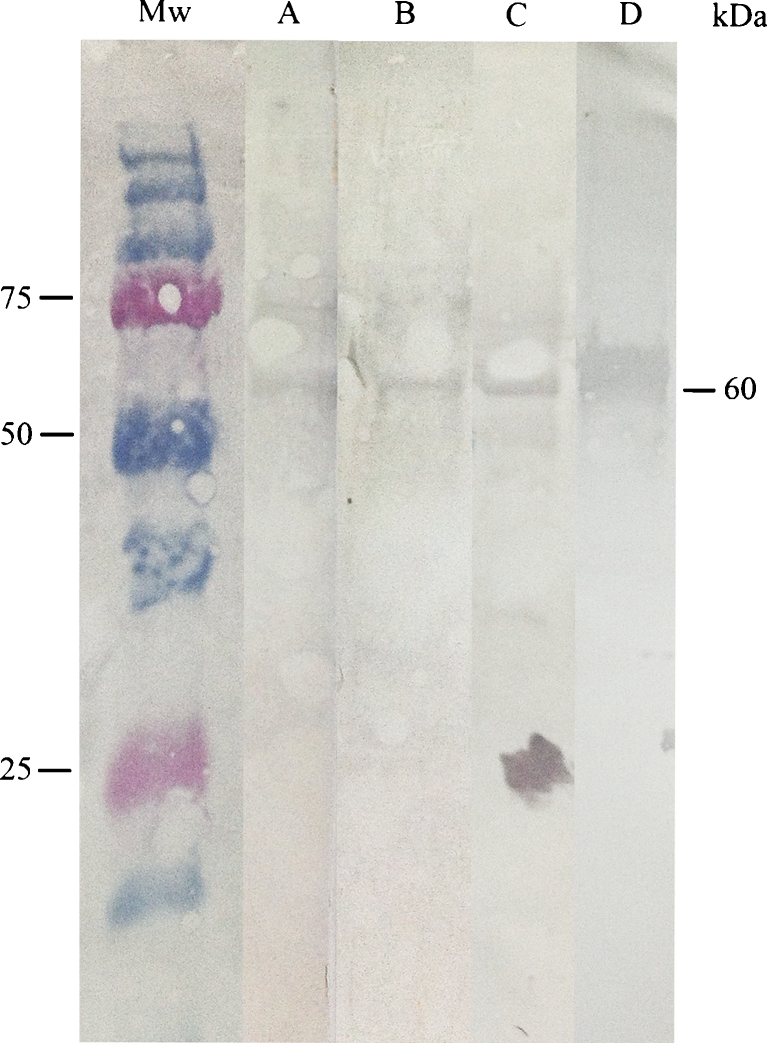
Western blot (WB) analysis of IgG antibodies against an antigenic peptide (*omp*B) of *Rickettsia helvetica*. *Lanes A*, *B* and *C* demonstrate the specific reactions for each serum sample (S2) for patient nos. 6, 14 and 15 in Study 1 against the protein in the 60-kDa region. *Lane D* shows the specific reaction between the antigenic peptide and polyclonal rabbit anti-serum

The serological results and details from the medical records on each patient are summarised in Tables 1 and 2. Of all patients analysed, 20/206 (9.7 %) had IgG and/or IgM antibodies to *Rickettsia* spp. equal to or higher than the cut-off titre of 1:64 (Table 1). All negative controls were negative. All but one patient had available medical records from the time of disease, and their data are summarised in Table 2. Seven of the patients were males and 13 were females. The median age was 54 years, range 20–74 years. Seven of the seroreactive patients (nos. 1, 6, 7, 9, 14, 15 and 19) showed seroconversion or significant rise of titre, indicating recent infection or current infection, and 13 patients had titres compatible with past infection, of which five patients (nos. 5, 8, 13, 17 and 18) were judged as probable infection. Eleven of the 19 *Rickettsia* spp. seroreactive patients were positive also for *Borrelia* spp. Six of the 19 were seroreactive for *Anaplasma* sp., of which five were serologically judged as having a probable infection and one was judged as positive. Five patients were seroreactive for all three agents (Table 2). None of the patients that were seroreactive for *Rickettsia* spp. were co-infected with TBE. Seven of the 19 patients had detectable antibodies only for *Rickettsia* spp., two of which had titres indicating recent infection, three compatible with past infection and two a probable infection. EM and/or rash for a period of 0–7 days were observed at all combinations of seroreactivity (Table 2). A total of 15 patients presented EM (between 5 and 15 cm in diameter). Nine of these 15 were co-infected with *Borrelia* spp. and/or *Anaplasma* sp., and six patients presenting EM had antibodies only against *Rickettsia* spp. The corresponding figures for the rash were nine *Rickettsia* spp.-reactive, of which six were co-infected (Table 2). Seventeen of the 19 *Rickettsia* spp.-reactive patients were tick-bitten 1–4 weeks earlier, two had suspected bites, five reported fever (>37.5 and <39 °C) lasting less than 1 week and 4 of 5 also experienced chills for a period of 0–3 days. Eight experienced headache lasting between 0 and 7 days. Five of 19 patients had muscle pain. Seven of 19 had respiratory symptoms, usually cough. All patients showed normal values for haemoglobin, white blood cell count, platelet cell count, alanine and aspartate aminotransferase, and lactate dehydrogenase in both serum S1 and S2. Three of the 19 patients were treated with doxycycline 100 mg once a day, the others with phenoximethyl penicillin. All patients except one (no. 7) were cured at follow-up after 2 months. Patient no. 7 showed persistent skin problems. WB for patient nos. 6, 14 and 15 showed a specific response to the mass-specific protein antigen in the 60-kDa region for IgG (Fig. 1).

**Table 1:** Antibody titres of serums 1 (S1) and 2(S2) for the 20 *Rickettsia* spp.-seroreactive patients in Study 1

Patient no.	S1		S2	
	IgG	IgM	IgG	IgM
1	<64	128	512	64
2	<64	<64	64	<64
3	128	128	64	<64
4	64	64	64	<64
5	<64	128	128	64
6	<64	<64	1,024	64
7	<64	64	256	128
8	<64	64	128	<64
9	<64	128	256	64
10	<64	128	64	<64
11	<64	256	<64	64
12	<64	256	64	64
13	<64	128	128	64
14	<64	128	512	<64
15	<64	128	512	<64
16	<64	128	64	<64
17	<64	128	128	64
18	64	<64	128	64
19	<64	128	512	<64
20	<64	128	64	<64

**Table 2:** Clinical symptoms, number of tick bites, treatment and results of serology for *Borrelia* spp. and *Anaplasma* sp. for the *Rickettsia* spp.-seroreactive patients in Study 1

Patient no.	Age/sex	Tick bite (no.)	Fever	Headache	Muscle pain	Rash	Respiratory symptoms	EM	*Borrelia* serology	*Anaplasma* serology	Treatment
1	76 M	ND	ND	ND	ND	ND	ND	ND	ND	ND	pc
2	70 F	Y	N	Y	N	N	N	Y	Neg	Neg	pc
3	59 F	Y	N	Y	N	Y	N	Y	Neg	Neg	pc
4	59 M	S	N	N	N	Y	N	Y	Neg	Neg	pc
5	57 F	Y	N	N	N	N	Y	Y	Neg	Neg	pc
6	57 F	Y	Y	Y	N	Y	Y	Y	Neg	Neg	pc
7	46 M	Y (>1)	Y	Y	Y	N	Y	N	Neg	Neg	pc
8	20 F	Y	N	N	N	N	N	Y	Neg	Neg	pc
9	74 M	Y	N	N	Y	Y	N	N	Pos	Pos	doxy
10	70 F	Y	N	N	N	Y	N	Y	Pos	Neg	pc
11	68 F	S	N	N	N	N	N	Y	Neg	P	pc
12	61 F	Y	N	Y	N	Y	N	Y	Pos	P	pc
13	57 F	Y	N	Y	N	N	Y	Y	Pos	Neg	pc
14	56 M	Y	N	N	N	Y	N	Y	Pos	Neg	pc
15	55 F	Y	N	N	N	N	N	Y	Pos	Neg	doxy
16	54 F	Y	N	N	N	Y	Y	Y	Pos	P	pc
17	52 M	Y (>1)	Y	Y	Y	N	N	Y	Pos	P	pc
18	51 M	Y (>1)	Y	Y	Y	N	Y	N	Pos	P	doxy
19	43 F	S	N	N	N	Y	N	Y	Pos	Neg	pc
20	26 F	Y (>1)	Y	N	Y	N	Y	N	Pos	Neg	pc

*ND* no data available; *Y* yes; *N* no; *S* suspected; *P* probable; *EM* erythema migrans; *doxy* doxycycline, *pc* penicillin

### Study 2

The serological results and the symptoms of the patients are summarised in Table 3. Sixteen patients were seroreactive for *Rickettsia* spp. Eleven of the 16 patients were primarily analysed for *Borrelia* spp., of which four (patients C, D, I and L in Table 3) were found to be seroreactive for *Rickettsia* spp., consistent with recent or current infection, and five of the 16 patients were primarily analysed for *M. pneumoniae*, of which one (patient P in Table 3) showed serological evidence of recent or current infection. The symptoms for each patient are shown in Table 3. The referring physicians had reported arthritis, rash, abdominal discomfort, muscle weakness, myalgia and prolonged cough. Cough and arthritis (including joint swelling, stiffness and pain) were predominant symptoms independent of the primary indication for sampling. In the control group of blood donors, all without clinical symptoms, one of the 80 patients had IgG 1:64 and IgM 1:64–1:128, respectively, interpreted as a previous exposure. Three of the 80 patients had only IgM between 1:64–1:128 and was non-reactive for IgG, probably as a result of non-specific reactivity or previous exposure. The re-testing of all seroreactive showed equivalent results.

**Table 3:** Antibody titres and symptoms for patients in Study 2

Patient	S1		*Borrelia* serology	M.p. serology	Symptoms
	IgG	IgM			
A	128	128	Neg	ND	EM
B	128	64	Neg	ND	Arthritis
C	256	1,024	Pos (IgG)	ND	EM
D	256	<64	Neg	ND	Abdominal
E	64	<64	Pos (IgG/M)	ND	Arthritis
F	<64	64	Pos (IgM)	ND	EM
H	<64	128	Neg	ND	Cough
I	512	512	Neg	ND	Cough, arthritis
J	32	256	Pos (IgG)	ND	Myalgia/tendinitis
K	64	128	Pos (IgG)	ND	Cough
L	256	<64	Neg	ND	Neuropathia
P	2,048	256	ND	Neg	Fever, cough
Q	128	64	ND	Neg	Fever, cough
R	<64	256	ND	Neg	Cough
S	64	64	ND	Pos (IgG)	Cough
T	64	128	ND	Neg	Abdominal

*M.p.* *Mycoplasma pneumoniae*; *ND* no data available; *EM* erythema migrans

## Discussion

This report presents the serological evidence of recent or probable rickettsial infection, in most cases, probable co-infection, in almost 6 % of patients from a prospective, clinical investigation (Study 1), where patients were recruited on the basis of EM and/or general signs of infection (fever, headache, muscle pain) following known or probable tick bite (Table 1). Three patients had IgG antibodies at the first visit, probably due to a past infection, and another 17 patients showed seroconversion or a 4-fold rise in IgG titre at the second visit, of which seven had a recent or current infection and ten a past infection, of which five were judged as probable infection. IgG and IgM antibodies normally appear 3–10 days after disease onset and peak after 3–4 weeks. Treatment within 2–5 days of disease onset may also inhibit antibody production. Seventeen of the 20 patients had only IgM titre at the first visit, while 10/20 were seronegative for IgM at the second visit, which shows the importance of paired sera for the analysis of both IgG and IgM antibodies to establish a reliable serological diagnosis. The specificity of the serological response showing the presence of IgG antibodies to *Rickettsia*-specific protein was demonstrated by WB analysis in three of the seroreactive samples (S2) (Fig. 1).

 The study also shows that *Rickettsia* spp. infection occurs either as a single infection or as a co-infection in patients with EM or serological evidence of *Borrelia* spp. or *Anaplasma* sp. infection. Of the 206 patients in the study, 174 were recruited on the basis of EM and 32 because of flu-like symptoms in combination with a preceding tick bite. All patients who were seroreactive for *Rickettsia* spp. in paired sera presented different disease symptoms comparable to those of LB (Table 2). The observed symptoms were similar and gave no guidance in relation to the causative agent. The variability of the clinical picture in LB has been highlighted in a recent report showing that asymptomatic *B. burgdorferi* infections, documented by seroconversion, were found more often than symptomatic infections in individuals bitten by a *B. burgdorferi*-infected tick [18]. No corresponding study has been carried out regarding *Rickettsia*, but in the present study, all patients with proven rickettsial antibodies presented varying degrees of symptoms. However, the symptoms of *Rickettsia* spp. or *B. burgdorferi* infection are, in both cases, quite general and do not allow us to distinguish between the agents.

 EM is typically regarded as a clinical sign consistent with LB [19]. Fifteen of the 19 *Rickettsia* spp.-seroreactive patients showed EM, of whom eight were serologically positive also for *Borrelia* spp. EM occurred also in six of the seven (6/206, 2.9 %) patients who were seroreactive only for *Rickettsia* spp., which may indicate that either the *Rickettsia* infection was causative or that Lyme infection did not produce antibody development. There are only a few previous reports in which EM or erythema resembling EM has been associated with rickettsial infection [20, 21]. Further serological examination including several agents in prospective clinical cases may provide more guidance as to the causative agent. Skin biopsy for PCR and/or immunohistochemistry and the detection of *Rickettsia* spp., *Borrelia* spp. and/or *Anaplasma* sp. organisms are also likely to be of value in clarifying the cause. Because several tick-borne agents give similar symptoms and, as the present study indicates, may occur as co-infections or separately, the task of providing a correct diagnosis is complex. In patients exposed to ticks presenting with unspecific symptoms, there is reason to consider the choice of antibiotics if other infectious agents are not excluded [14]. If EM is the only found symptom and causes other than LB can be excluded, it is likely that phenoxymethylpenicillin is sufficient as the drug of choice. Thus, serological testing is important for a full clinical assessment of underlying causes.

 Study 2 gives a similar picture for 16 seroreactive patients with symptoms involving the skin, joints (culture-negative arthritis), headache and cough. The reason for the cough symptom requires further study, but it is known that rickettsiosis gives pulmonary vasculitis, which could be a possible explanation.

 Screening for *Rickettsia* spp. from different localities in Sweden, using PCR, has demonstrated a mean infection prevalence of 1.5–17.3 % for *R. helvetica* in *I. ricinus* ticks, including all stages, proving that Sweden is an endemic area for this agent and that the risk of infection is consistent with the tick’s distribution [4].

 Previous smaller serosurveys in Sweden have shown IgG antibodies to *Rickettsia* spp. in the serum of up to 4.4 % of tick-exposed subjects, compared to 0.6 % in healthy blood donors [15]. In a prospective study of Swedish recruits who trained in the coastal areas, 8.9 % showed seroconversion compared to the proportion of 9.2 % found to be seroreactive in France in a group of forestry workers, and in a study from Laos, 2.9 % of adults admitted to hospital because of fever showed seroconversion to *R. helvetica* [5, 6, 22, 23].

 The current more extensive study demonstrates both seroconversion and significant rise of titres for *Rickettsia* spp. in single-infected individuals as well as in those patients co-infected with other tick-borne agents known to present similar clinical symptoms. The complexity of the clinical picture needs to be considered when diagnosing the causative agent and selecting appropriate treatment. It also demonstrates a need for further studies.
